# ATR-FTIR Spectroscopy, a New Non-Destructive Approach for the Quantitative Determination of Biogenic Silica in Marine Sediments

**DOI:** 10.3390/molecules24213927

**Published:** 2019-10-31

**Authors:** Dora Melucci, Alessandro Zappi, Francesca Poggioli, Pietro Morozzi, Federico Giglio, Laura Tositti

**Affiliations:** 1 Department of Chemistry “G. Ciamician”, University of Bologna, 40126 Bologna, Italy; alessandro.zappi4@unibo.it (A.Z.); francesca.poggioli3@studio.unibo.it (F.P.); pietro.morozzi2@unibo.it (P.M.); laura.tositti@unibo.it (L.T.); 2 Polar Science Institute-National Research Council ISP-CNR, Via P. Gobetti 101, 40129 Bologna, Italy

**Keywords:** diatoms, biogenic silica, ATR-FTIR, chemometrics, NAS

## Abstract

Biogenic silica is the major component of the external skeleton of marine micro-organisms, such as diatoms, which, after the organisms death, settle down onto the seabed. These micro-organisms are involved in the CO_2_ cycle because they remove it from the atmosphere through photosynthesis. The biogenic silica content in marine sediments, therefore, is an indicator of primary productivity in present and past epochs, which is useful to study the CO_2_ trends. Quantification of biosilica in sediments is traditionally carried out by wet chemistry followed by spectrophotometry, a time-consuming analytical method that, besides being destructive, is affected by a strong risk of analytical biases owing to the dissolution of other silicatic components in the mineral matrix. In the present work, the biosilica content was directly evaluated in sediment samples, without chemically altering them, by attenuated total reflection Fourier transform infrared (ATR-FTIR) spectroscopy. Quantification was performed by combining the multivariate standard addition method (MSAM) with the net analyte signal (NAS) procedure to solve the strong matrix effect of sediment samples. Twenty-one sediment samples from a sediment core and one reference standard sample were analyzed, and the results (extrapolated concentrations) were found to be comparable to those obtained by the traditional wet method, thus demonstrating the feasibility of the ATR-FTIR-MSAM-NAS approach as an alternative method for the quantification of biosilica. Future developments will cover in depth investigation on biosilica from other biogenic sources, the extension of the method to sediments of other provenance, and the use higher resolution IR spectrometers.

## 1. Introduction

In the present work, we introduce an innovative and non-destructive method for the quantification of biogenic silica in marine sediments through the use of infrared spectroscopy combined with chemometrics. The proposed method was applied to sediment samples coming from Terra Nova Bay, Antarctica.

Antarctica is a unique natural laboratory because it is the coldest, driest, highest, windiest, and most isolated continent. Therefore, it is almost unaffected by anthropogenic influence [1]. The Southern Ocean allows the diffusion of atmospheric carbon dioxide into the deep sea, which is partially used by sea plants for growth and for the production of organic matter [2]. Therefore, this region is one of the most important for the study of climate changes and conditions of the ocean [3]. 

In particular, an important tool to control the chemical composition of seawater and to reconstruct paleo-ocean conditions is represented by marine sediments, which are a reservoir and a sink of chemical species involved and cycled in the marine food chain [1]. Among nutrients, silicon is an essential element in the ocean ecosystems, because it is responsible for the growth of Radiolaria, Sponges, Phaeodaria, and particularly Diatoms, which represent a major portion of planktonic primary producers [4]. Diatoms are planktonic unicellular microalgae, known to form an external skeleton called frustule, constituted by amorphous silica and organic components (usually including long-chain polyamines and silaffins) [5,6]. After their death, the diatom siliceous skeleton settles down through the water column. The extent of diatom deposition in the sediments will be a function of the sea bottom depth and of the degree of solubilization of opal silica in the water column [7]. Siliceous microfossils, therefore, can represent a large part of the mass of biogenic sediments accumulating on the deep-sea floor [8]. 

In the whole Southern Ocean, the Ross Sea is the region of the most widely extensive algal blooms, usually initiating in the Ross Sea polynya [9], an ice-free area of enhanced bio-productivity that can be considered as a biological “hot spot” compared with the surrounding waters. This area extends to the open sea surface as soon as the austral summer develops and the sea ice melts [10,11]. It plays a key climatic role on a global scale. Indeed, the Ross Sea is one of the main sink areas for the tropospheric CO_2_, widely contributing to counterbalancing its budget and the associated role in climate change [12,13]. In the western Ross Sea, the polynya of Terra Nova Bay (TNB) is an area of high accumulation of biogenic silica in the sediments [14,15].

Biogenic silica (BSi) content in marine sediment can be considered as a good proxy to characterize the bio-productivity of the Southern Ocean [16,17]. However, the quantification of BSi is complicated by the presence of lithogenic silica, which is chemically equivalent to BSi (SiO_2_), with the only difference being crystalline, while BSi is amorphous. Several methods have been proposed to estimate BSi in marine sediments: (1) X-ray diffraction after the conversion of opal to cristobalite at a high temperature [18]; (2) direct X-ray diffraction of amorphous silica [19]; (3) direct infrared spectroscopy of amorphous opal [20]; (4) elemental partitioning of sediment chemistry [21,22]; (5) microfossil counts [23,24]; and (6) several wet-alkaline extraction methods [7,25,26]. 

Among the above-mentioned techniques, the wet alkaline methods are the most popular because they are the most sensitive techniques for BSi assessment. According to these methods, BSi is extracted and distinguished from lithogenic silica based on hot alkaline solutions [7]. Wet methods exploit a different rate of dissolution of lithogenic and biogenic silica in alkaline solution, with BSi dissolving more quickly than the mineral component. Solubilized BSi can, therefore, be collected in the supernatant of the solution, and subsequently determined by spectrophotometry. Such separations are extremely demanding and time-consuming, and above all, they do not ensure the quantitative recovery of BSi, owing to inherent systematic problems; that is, dependence on matrix effects, incomplete opal recovery, and contamination by non-biogenic silica [17,23].

The increasing success of chemometric tools applied to basic spectrophotometric techniques such as Fourier transform infrared (FTIR), together with the compelling need for understanding key biogeochemical processes of global importance, have recently inspired the introduction of an alternative approach to solve the problem of BSi assessment. In particular, FTIR spectroscopy has been applied to lacustrine sediments for the analysis of silica and other minerals by Rosén et al. [27,28]. Vogel et al. and Rosén et al. [29,30] showed that FTIR spectroscopy in the mid-infrared region is highly sensitive to chemical components present in minerogenic and organic material, such as sediments; this fact provides an efficient tool for quali- and quantitative characterization of these fundamental, but complex environmental matrices.

Moreover, a method based on attenuated total reflectance (ATR)-FTIR measurements has also been proposed in the literature to quantify inorganic components in marine sediments [31,32]. ATR-FTIR spectroscopy is particularly appealing for the analysis of sediments because no chemical sample pre-treatment is required: it may in principle by-pass all the drawbacks of the wet-chemical method; moreover, it works with small amounts of sample material (0.05–0.1 g, dry weight) and it is rapid, inexpensive, and efficient. Besides, ATR-FTIR is a non-destructive method, allowing to recover the sample for further analyses, and it can be carried out even off the lab. 

In the present work, we developed and present an analytical method based on ATR-FTIR for the quantitative determination of biosilica content in marine sediments. The feasibility of the method was evaluated by quantifying BSi in a series of sediment samples collected in the Ross Sea. Optimization of the experimental procedures such as the drying process, homogenization, and deposition of the sample on the ATR crystal are discussed in detail, in order to provide a reliable background useful to solve reproducibility problems, which may constitute a drawback of such a simple instrumental approach. Furthermore, the strong matrix effect intrinsic to environmental samples is faced and solved by applying a multivariate standard addition method (MSAM) [33], improved by net analyte signal (NAS) computation [34,35].

## 2. Results and Discussion

### 2.1. ATR Spectra

For each of the 22 analyzed sediment samples (21 coming from Mooring D and one 53%*_w_*_/*w*_ reference standard), four standard-added (add.*x*) samples were prepared: the zero-added sample (add.0) is the pure sample, add.1 has an added concentration of diatomite at 5%*_w_*_/*w*_, add.2 at 10%_*w*/*w*_, and add.3 at 15%_*w*/*w*_. All added samples (and a pure diatomite sample) were analyzed by ATR-FTIR.

In the ATR spectra of marine sediments, the contribution of silica, both biogenic and lithogenic, is dominant. Such spectra exhibit four characteristic vibrational bands. The two main bands at 1100 and 471 cm^−1^ are attributed to triply degenerated stretching and bending vibration modes, respectively, of the [SiO_4_] tetrahedron [36]. The band at 800 cm^−1^ corresponds to an inter-tetrahedral Si–O–Si bending vibration mode, and the band near 945 cm^−1^ to an Si–OH vibration mode [37]. Previous studies have shown that the absorbance centered around 1640 cm^−1^ and between 3000 and 3750 cm^−1^ can be attributed to hydroxyl vibrations because hydroxyl ions are major constituents of clay minerals, opal, and organic compounds present in marine sediments [38]. However, these bands are not specific for silica, their intensity is generally low (about one-tenth of the main band); moreover, they are overlapped with the residual absorption bands of H_2_O. Therefore, to reduce the noise in the spectral data acquired, we decided to discard the IR region between 4000 and 1300 cm^−1^ and to apply the chemometric procedure only in the region between 1300 and 400 cm^−1^.

**Figure 1:**
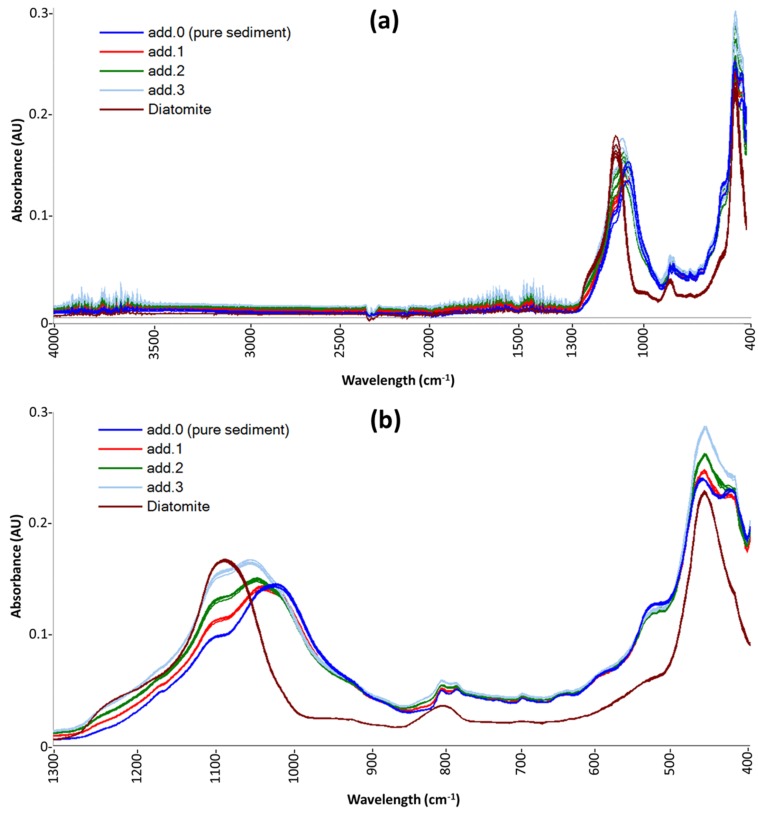
(**a**) ATR raw spectra of sample D10, as obtained from the spectrophotometer; (**b**) the same spectra after band removal (discarding the IR range 4000–1300 cm^−1^) and after multiplicative scatter correction (MSC) pre-treatment. “add.” in the legends indicates standard added samples.

Figure 1 shows the raw spectra of sample D10 (as an example of the spectra obtained for all sediment samples) and the replicates of a pure-diatomite sample. In Figure 1a, spectra obtained by instrumental analysis are shown, while Figure 1b highlights the effect of the spectral pre-treatments: uninformative-band removal and MSC.

On the spectra reported in Figure 1b, the two chemometric procedures described in Section 3.4 were carried out for all sediment samples, and the results are reported in Table 1. The expected values reported in Table 1 are the BSi concentrations obtained by wet analyses that were carried out only on five sediment samples (and on 53%*_w_*_/*w*_-standard): D4, D6, D9, D18, and D21.

### 2.2. Results: Procedure 1

To assess the reliability of the new methodology proposed here, the BSi results obtained here can be compared to those achieved through the traditional wet method, taken as a reference. Table 1 shows that the results obtained with *Procedure 1* (band removal and MSC) are in good agreement with the expected values obtained when samples were analyzed with the wet method. Indeed, the confidence intervals obtained for these five sediment samples by NAS are not significantly different from the ones obtained by the wet method (at 0.05 significance). Also, the result obtained for the standard sample (Std 53%*_w_*_/*w*_) is in agreement with the expected concentration. The coefficients R^2^ of the NAS standard addition lines are all higher than 0.98, indicating a good correlation between added concentrations (dependent variable) and the pseudo-univariate NAS values calculated by the chemometric procedure. The LoD values are also very good, being, in general, in the order of magnitude of one-tenth (or even lower) with respect to the corresponding extrapolated BSi concentration. 

Moreover, Frignani et al. [15] reported that BSi concentration in surface sediments in this area is usually relatively low, <10%*_w_*_/*w*_; the results obtained by NAS are in agreement with this consideration, as D0, D1, D2, and D3 extrapolated concentrations are lower than the indicated value.

All these considerations confirm that NAS applied to ATR spectra of the standard added samples can be a valuable and reliable alternative to the time-consuming wet method for the quantification of BSi in marine sediments.

The main drawbacks concern the three samples for which no results were obtained: D12, D13, and D16. In these cases, the NAS standard addition lines had, for all PLS-factors, either a negative slope or intercept, giving negative extrapolated values, or not acceptable R^2^ (lower than 0.7), that make any possible result unreliable. The reason for such behavior is still under study, but we can hypothesize that there is still some source of noise in ATR analysis that was not taken into account, although several precautions were taken during instrumental analyses, as described in Section 3.3. We, therefore, decided to proceed with further chemometric assessments, also to test the hypothesis of a possible defect in the NAS procedure.

### 2.3. Results: Procedure 2

**Figure 2:**
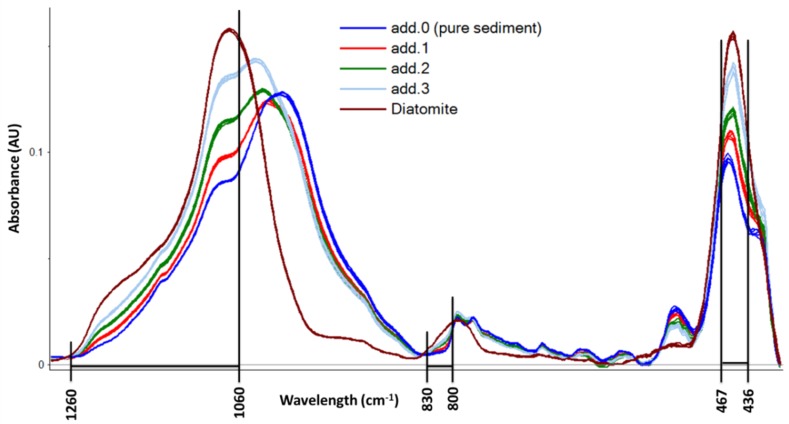
Baseline-corrected ATR spectra of sample D10. Black lines indicate the variables considered most important by partial least square (PLS) correlation loadings.

As described in Section 3.4, a variable selection was carried out on baseline-corrected spectra. Correlation loadings on PLS-factor 1 were used to select the most important variables to describe the regression model. Although a different variable selection was carried out for each sediment sample, not always giving the same variables, a general description of the selected IR bands can be drawn and is resumed in Figure 2. High correlation loading values in the PLS-factor 1 are computed in the regions of 1260–1060 cm^−1^, 830–800 cm^−1^, and 467–436 cm^−1^. These regions of the IR spectra correspond to the characteristic SiO_2_ absorbance maxima as reported by Vogel et al. [29]. On these selected variables, NAS computation was carried out and the results are reported in the last vertical section of Table 1.

Again, concentrations extrapolated by NAS are not significantly different from the “wet method-based” values, with high R^2^ (>0.95). After the application of this second procedure (baseline correction and variable selection before NAS), significant differences were detected only for D0 and D17, which, in this case, also have a lower R^2^ compared with the other samples. In this case, some problems arise from LoDs, which, in most cases, are comparable to the extrapolated value (and also higher than that for D20). Such a drawback might, therefore, be because of the spectral pre-treatment; in order to calculate LoD, a blank spectrum is necessary. However, such a blank spectrum has to be pre-treated as all the other spectra, and in this case, it has to be baseline corrected. In this way, the pre-treatment can likely produce some spikes in the blank spectrum (that is, a noisy signal oscillating around the zero), thus affecting the computation of LoD.

The three samples that did not give results with the computation by *Procedure 1* (D12, D13, and D16) in this case have an acceptable extrapolated concentration. However, there are again three samples (D1, D7, and D8) with no result. This strengthens the hypothesis of the presence of a noise source that was not taken into account. Indeed, variable selection may reduce the noise present in the whole spectrum, but, at the same time, if noise is present in the selected variables, its effect may be enhanced. Therefore, the two chemometric methods presented in this work may be considered to be complementary for this study.

## 3. Materials and Methods 

### 3.1. Study Area

**Figure 3:**
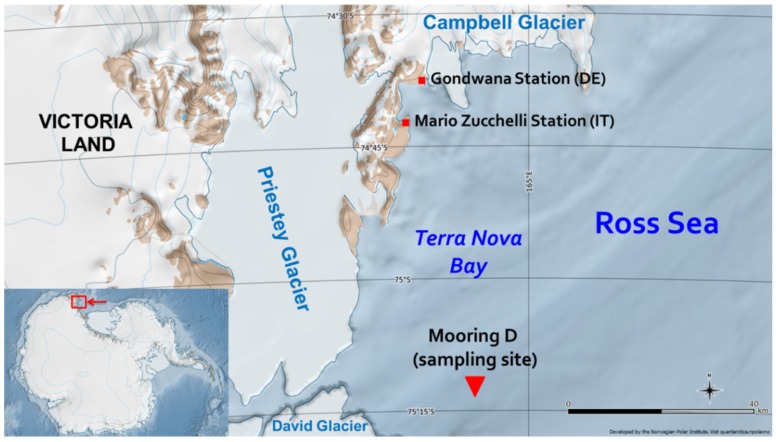
Sampling site (Mooring D) in Terra Nova Bay, Antarctica.

Sediment samples for the present study were collected in “mooring D” (or “site D”), which is located in Antarctica, in the western sector of the Ross Sea continental shelf within the polynya of Terra Nova Bay at 75°06′ S and 164°28′5′′ E (Figure 3). The box-core, from which the sediments were collected, was sampled at a depth of 972 m during the 2003–2004 Italian PNRA (Programma Nazionale di Ricerca in Antartide) Campaign [39], whose basis was situated in the “Mario Zucchelli” station. 

In the Ross Sea, surface sediments are generally composed of unsorted ice-rafted debris, terrigenous silts and clays, and siliceous and calcareous biogenic debris [40]. In site D, in particular, coarse terrigenous deposits are predominant, owing to the proximity of Priestley, David, and Campbell glaciers [15]. 

### 3.2. Samples

The sediment collected in site D was sampled using a 1T Oceanic box corer. A sub core 22 cm long was collected by means of a polyvinyl chloride (PVC) liner. The short core was subsampled with a resolution of 1 cm [39]. Twenty-two sediment sections were thus obtained and named with a two-digit code: a letter, “D”, indicating the sampling place; and a number, from 0 to 21, indicating the core height, with D0 being the top, corresponding to the sediment surface. Sediments were then stored at −21 °C in a polycarbonate Petri capsule and oven-dried at 50 °C just prior to the analyses. The BSi content of five of these samples was also quantified by a wet method analysis, according to the DeMaster method [7,15], thus providing some comparison values for the ATR analyses. In the absence of a commercial certified reference material for BSi, the “internal reference standard” used in the Polar Science Institute-National Research Council (CNR-ISP) laboratory was adopted for the purpose of this paper. This sample consists of an Antarctic marine sediment analyzed repeatedly both in CNR and other biogeochemical laboratories, resulting in a BSi content of (53 ± 3)%*_w_*_/*w*_ and a remaining 47%*_w_*_/*w*_ of alkaline halide.

**Figure 4:**
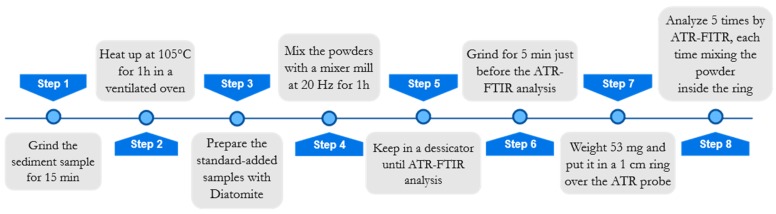
Sample preparation flowchart. ATR-FTIR, attenuated total reflection Fourier transform infrared.

**Figure 5:**
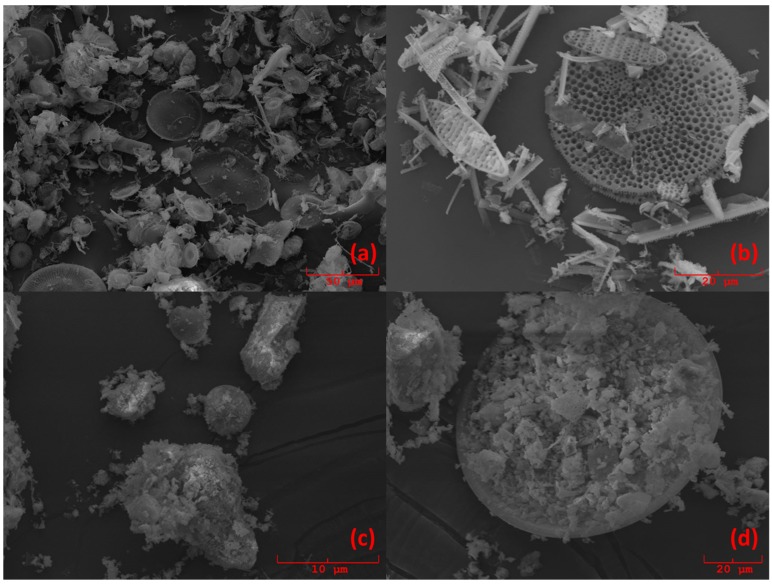
Scanning electron microscope (SEM) images of samples (**a**) pure diatomite; (**b**) 53% standard; (**c**) D1 sample, which is characterized by the presence of both radiolaria and bulks of sedimentary material; and (**d**) D4 sample.

For the sake of readability, a flowchart concerning the sample preparation is reported in Figure 4. Before sample preparation, all samples were manually ground in an agate mortar for approximately 15 min and heated in a ventilated oven at 105 °C for 1 h to remove atmospheric moisture. Afterward, each sample was split into four aliquots, three of which were added with known amounts (5%*_w_*_/*w*_, 10%*_w_*_/*w*_, 15%*_w_*_/*w*_) of Diatomite (Celite^®^ 545 AW, Sigma-Aldrich, Darmstadt, Germany), in order to apply the multivariate standard addition method. The total weight of each standard-added sample was 200 mg. Diatomite was chosen as a proxy of standard biogenic silica, because it is composed of frustulae of biogenic silica, similar to what we wanted to quantify in marine sediments. Such a similarity was visually evaluated by analyzing some samples with a scanning electron microscope (SEM) Philips 515B (Philips, Amsterdam, Netherlands), equipped with an EDAX DX4 microanalytical device (EDAX Inc., Mahwah, NJ, USA). Figure 5 shows the pictures obtained by SEM. From Figure 5c,d, it can be seen that samples D1 and D4 contain the same radiolaria present in the Diatomite (Figure 5a) used as a proxy of BSi.

To ensure better homogenization of the powders, a Mixer Mill “MM20” (Retsch Inc., Düsseldorf, Germany) was used. Each added sample was placed in stainless steel cylinders of 1.5 mL volume and left in the ball mill for 60 min at 20 Hz. Before the instrumental analysis, samples were kept in a desiccator filled with silica gel to prevent the absorption of atmospheric moisture. Standard added samples were then analyzed by ATR-FTIR spectroscopy. 

### 3.3. ATR-FTIR Analysis

Attenuated total reflection spectra were collected using a Bruker ALPHA FT-IR spectrometer (Brucker Optics GmbH, Billerica, MA, USA) equipped with a single-reflection diamond ATR accessory (Bruker Platinum ATR, Billerica, MA, USA) with an approximately 0.6 mm × 0.6 mm active area and a mercury–cadmium–telluride detector. Spectra were collected in the mid-IR range, 400–4000 cm^−1^, with an optical resolution of 4 cm^−1^; the registered spectrum is the mean of 64 scans, executed in 3 min. For each sample aliquot, five replicate spectra were recorded to assess precision and ensure the reproducibility of each sample. All measurements were performed at ambient conditions. Before spectra acquisition, a background spectrum (air) was collected with the same operational parameters. Such a background was automatically subtracted to each sample spectrum.

To optimize the analytical reproducibility, some precautions were taken for ATR analysis. Indeed, it is widely reported in the literature how an imprecise sample preparation (especially drying process), sample deposition, and instrumental calibration may cause poor instrumental repeatability and accuracy, fundamental characteristics for quantification purposes [31,41]. For these reasons, a suitable experimental protocol was developed and evaluated (Figure 4). 

Before the instrumental analysis, samples were manually ground again for 5 min in an agate mortar, in order to homogenize powder granulometry. Moreover, for each added sample, an aliquot (53 mg) was carefully weighted and lodged in a steel ring of 1 cm in diameter, which was then placed over the spectrophotometer probe. The same amount of material was taken for all the analyzed samples, to maximize reproducibility and reduce scattering and other problems resulting from not optimal (or not constant) contact between the sample and crystal. These problems become relevant in ATR analysis when used for quantification purposes owing to the geometry of ATR irradiation and reflectance, which need an accurate evaluation and the adoption of a suitable experimental protocol [42]. 

### 3.4. Chemometrics

Prior to chemometric analysis, the five replicated spectra of each added sample were pre-treated by multiplicative scatter correction (MSC) [43]. MSC allows the reduction of the effects of scattering noise on IR spectra, increasing the reproducibility of sample replicates.

Subsequently, in order to calculate the BSi content in each sediment sample by MSAM, the NAS procedure was applied [35,44]. NAS is a mathematical procedure that allows extracting, from a multivariate signal (in this case, an ATR spectrum), that part of the signal that is only due to the analyte, removing the other signals due to the other interfering species present in the matrix [35]. In this way, the multivariate problem can be reduced to a pseudo-univariate problem, whose results can be obtained by a univariate treatment. NAS computations were performed as follows.

The NAS procedure starts from a partial least square (PLS) regression [45], using the ATR spectra as independent variables (*X*) and the added concentrations vector as dependent one (*y*). The best PLS-factor (*A*) has to be selected and the corresponding PLS-regression coefficient vector (*b_A_*) is used to compute a projection matrix (*H*) as follows:

(1) $$H=b_{A}{\left(b_{A}b_{A}^{t}\right)}^{-1}b_{A}^{t},$$

where *t* indicates transpose and superscript “−1” indicates matrix inversion. *H* matrix is then used to compute NAS vectors ($x_{i}^{*}$):

(2) $$x_{i}^{*}=Hx_{i},$$

where *x_i_* are the rows of matrix *X*, which means samples of ATR spectra. Each calculated x_i_* corresponds to the net signal (devoid of interfering signals) of each replicate of the added samples. The Euclidean norms of such net signals can be then used as pseudo-univariate signals to compute a univariate standard addition linear regression line, from which BSi concentration can be obtained by extrapolation.

The selection of the optimal PLS-factor (*A*) is a crucial point of the procedure, because, in most of the cases, the final extrapolated concentration varies (also dramatically) while varying *A*. Therefore, *A* was chosen (sample by sample) as the PLS-factor that optimizes both the PLS root mean squared error (RMSE), by minimizing it, and the determination coefficient (R^2^) of the final pseudo-univariate line, by maximizing it. When these two conditions were not simultaneously achievable for one PLS-factor, *A* was chosen as the factor giving the best compromise between these two parameters, based on the highest R^2^.

The standard deviations of the extrapolated concentration values were computed by the *jackknife* method [46]. Once the optimal PLS-factor is selected, the *jackknife* procedure replicates the NAS computation as many times as the number of objects (*x_i_*), each time keeping out one object. In this way, *i* different extrapolated values are obtained for each NAS computation and the overall standard deviation is estimated as the standard deviation of the *jackknife*-extrapolated values.

Limits of detection (LoDs) were computed collecting five replicates (the same number of the other samples) of a blank spectrum (empty sample holder) and projecting them onto the NAS space by Equation (2) as if they were real samples [47]. The so obtained NAS-blank signals were mediated to obtain the vector *ε*, and the LoD was computed as follows [47]:

(3) $$\mathrm{LoD}=3\frac{\left\|\varepsilon \right\|}{\left\|b_{A}\right\|},$$

where ‖·‖ indicates the Euclidean norm.

The so far described procedure (*Procedure 1*) was applied to raw spectra, as they were obtained from the spectrophotometer. This procedure gave reasonable results for the majority of the samples, while it failed for three of them; in those cases, for all PLS-factors, the final NAS standard addition line had either a negative slope or intercept, producing a negative extrapolated concentration. The reason behind such behavior is still under evaluation. In order to obtain a result for each sediment sample, another chemometric procedure (*Procedure 2)* has thus been developed. Instead of using raw spectra, a baseline correction was applied directly by the software controlling the instrument, OPUS v.7.2 (Bruker). MSC was applied to baseline-corrected spectra and, before NAS computation, a variable selection was applied. For variable selection, another PLS regression was computed (previously to the one used for NAS). Only factor 1, always retaining more than 95% of the explained variance, was considered, and the variables giving correlation loadings [48] higher than 0.7 (in absolute value) were retained as important. NAS computation was then applied only with these selected variables. The standard deviations on the extrapolated values and LoDs were calculated as before.

MSC and variable selection pre-processing were performed by the software The Unscrambler v.10.3 (CAMO, Olso, Norway), while NAS and *jackknife* procedures were computed by a homemade code in R environment (R Core Team, Vienna, Austria).

## 4. Conclusions

In this study, we demonstrated the feasibility of a new approach for the quantification of biogenic silica based on IR spectroscopy coupled with chemometrics. Biogenic silica content in marine sediments from Terra Nova Bay in West Antarctica was evaluated with Fourier-transform infrared spectroscopy in attenuated total reflection mode (ATR-FTIR). For quantification, the multivariate standard addition method (MSAM) was applied, and the net analyte signal (NAS) procedure was used to solve the problems deriving from the strong matrix effect affecting such analyses.

Twenty-one subsequent core samples and one reference standard were analyzed. Reliable results were obtained, as observed from the comparison with homologous data from the traditional wet method.

Some drawbacks remain. The chemometric procedure did not give acceptable results for some samples, even if a variable selection was carried out. Moreover, the limits of detection, and perhaps also standard deviations, are in some cases still too high.

However, it has to be taken into account that the quantification of biosilica, in this work, has been carried out with an analytical technique (ATR) that has several intrinsic drawbacks when performing quantitative analysis. In particular, owing to the optical behavior of photons at such low angles as in ATR, extremely careful handling of samples and highly reproducible sample geometry are required when analyzing powdered samples. Moreover, the analyzed samples are powders, which, despite all the precautions taken before and during the analysis, can still have some problems concerning homogeneity and granulometry. The BSi content was also evaluated in natural samples without any chemical pre-treatment, thus its analytical signal may be strongly affected by the presence of lithogenic silica, besides all the other species composing the sediments.

Considering all these aspects, the analytical and chemometric procedure presented in this work, although requiring some more refinements, can be considered a promising alternative to the traditional time-consuming wet method for the quantification of biosilica in marine sediments. In paleolimnological research, the ATR-FTIR technique is seldom used. The results presented here, as well as the fact that this method is fast and cost-effective, requiring only small quantities of sediment sample, should encourage more researchers to use it. Moreover, marine sediments are precious samples, which are difficult to collect; thus, a not-destructive method would be preferable to analyze them, although ATR-FTIR cannot yet entirely replace conventional analytical tools in paleolimnology.

## Figures and Tables

**Table 1:** Net analyte signal (NAS) results for the two pre-processing methods. All the numbers are formatted with three significant digits to allow for a detailed comparison. LoD, limit of detection.

		*Procedure 1*				*Procedure 2*			
SAMPLE CODE	Expected Value ± Standard Deviation (%*_w_*_/*w*_)	NAS Extr. C (%*_w_*_/*w*_)	Standard Deviation	R^2^	LoD	NAS Extr. C (%*_w_*_/*w*_)	Standard Deviation	R^2^	LoD
**Std 53%**	53 ± 3	53.6	6.02	0.992	1.01	50.2	2.74	0.988	7.76
**D0**	-	3.23	0.903	0.996	0.0265	8.64	3.01	0.956	2.39
**D1**	-	3.24	1.92	0.993	0.190	-	-	-	-
**D2**	-	9.88	1.70	0.994	0.416	9.28	1.23	0.990	6.24
**D3**	-	7.55	0.315	0.993	0.743	8.40	0.436	0.999	4.08
**D4**	13 ± 2	12.0	1.16	0.988	0.469	12.8	2.01	0.988	3.58
**D5**	-	5.41	1.10	0.993	0.0214	5.38	1.75	0.995	2.88
**D6**	14 ± 2	14.2	1.54	0.989	0.00946	14.2	2.71	0.997	1.41
**D7**	-	5.87	1.27	0.999	0.646	-	-	-	-
**D8**	-	9.45	0.671	0.993	0.380	-	-	-	-
**D9**	8 ± 1	8.26	3.16	0.986	0.654	8.91	0.514	0.982	4.68
**D10**	-	12.0	1.18	0.997	0.515	12.1	2.12	0.995	0.267
**D11**	-	2.94	0.646	0.994	0.0666	3.84	0.803	0.993	1.76
**D12**	-	-	-	-	-	10.6	2.32	0.995	2.32
**D13**	-	-	-	-	-	3.72	1.81	0.987	3.82
**D14**	-	9.00	0.146	0.998	0.133	10.9	0.433	0.998	7.62
**D15**	-	3.25	0.184	0.993	0.0784	5.19	0.122	0.996	2.22
**D16**	-	-	-	-	-	5.63	1.38	0.989	3.60
**D17**	-	2.71	0.535	0.994	0.0647	13.7	1.41	0.984	1.93
**D18**	4.2 ± 0.6	4.80	1.67	0.996	0.0900	5.16	1.70	0.992	4.34
**D19**	-	4.04	0.332	0.998	0.0459	3.22	0.345	0.997	1.23
**D20**	-	2.36	0.535	0.999	0.610	4.26	1.19	0.991	8.46
**D21**	3.4 ± 0.5	3.12	1.86	0.998	0.653	4.12	0.296	0.993	3.93
